# The genome sequence of black horehound, *Ballota nigra* L. subsp. *foetida* (Lam.) Hayek (Lamiaceae)

**DOI:** 10.12688/wellcomeopenres.19763.1

**Published:** 2023-10-12

**Authors:** Maarten J. M. Christenhusz, Michael F. Fay, Ilia J. Leitch, Royal Botanic Gardens Kew Genome Acquisition Lab, Darwin Tree of Life Barcoding collective, Plant Genome Sizing collective, Wellcome Sanger Institute Tree of Life programme, Wellcome Sanger Institute Scientific Operations: DNA Pipelines collective, Tree of Life Core Informatics collective, Darwin Tree of Life Consortium

**Affiliations:** 1 Royal Botanic Gardens Kew, Richmond, England, UK

**Keywords:** Ballota nigra, black horehound, genome sequence, chromosomal, Lamiales

## Abstract

We present a genome assembly from a specimen of *Ballota nigra* (black horehound; Tracheophyta; Magnoliopsida; Lamiales; Lamiaceae). The genome sequence is 1186.8 megabases in span. Most of the assembly is scaffolded into 11 chromosomal pseudomolecules. Three mitochondrial chromosomes were assembled, with lengths of 148,17, 121,67 and 125,74 kilobases. The chloroplast genome has been assembled and is 151.91 kilobases in length.

## Species taxonomy

Eukaryota; Viridiplantae; Streptophyta; Streptophytina; Embryophyta; Tracheophyta; Euphyllophyta; Spermatophyta; Magnoliopsida; Mesangiospermae; eudicotyledons; Gunneridae; Pentapetalae; asterids; lamiids; Lamiales; Lamiaceae; Lamioideae; Marrubieae; *Ballota*; *Ballota nigra* L. *Ballota nigra* subsp. *foetida* (Vis.) Hayek (NCBI:txid2561859).

## Background

**Figure 1.**
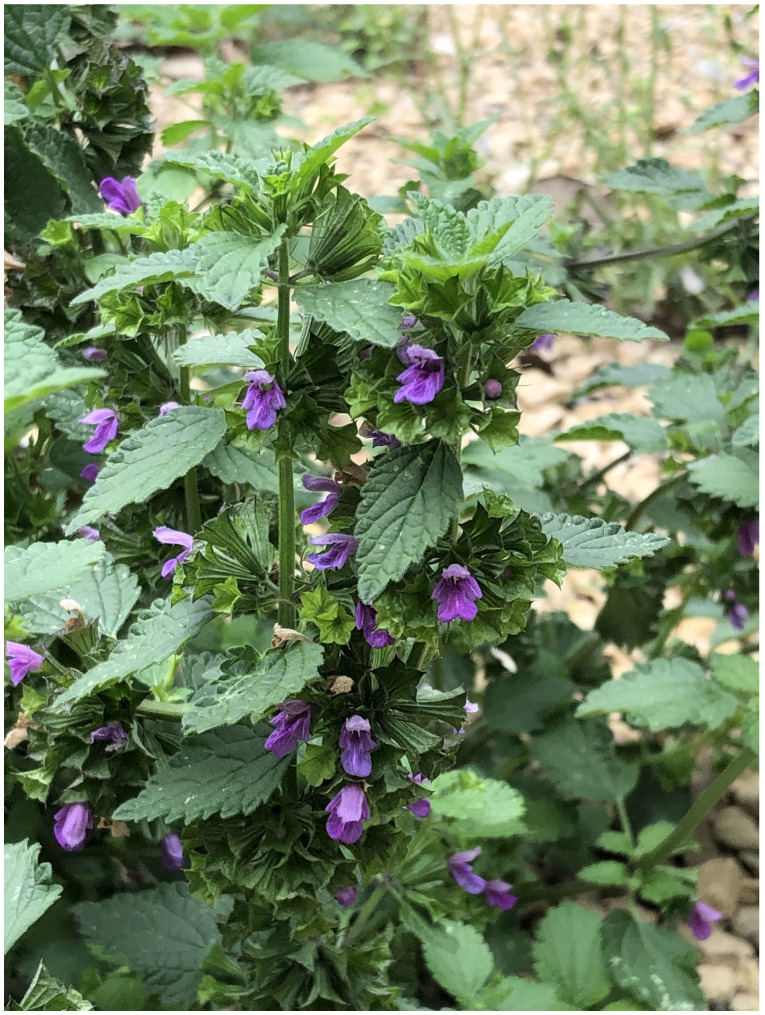
*Ballota nigra* subspecies *foetida* individual (daBalNigr1) sampled for the Darwin Tree of Life.

*Ballota nigra* L. (Lamiaceae), commonly known as the black horehound, is a variable species with eight currently accepted subspecies ( POWO, 2023). The plants growing in Britain and Ireland belong to *B. nigra* subsp. *foetida* (Vis.) Hayek, and are densely branched herbaceous perennials growing up to 80 cm tall with purple (sometimes pink or white) bilabiate flowers and dark green hoary leaves that emit a foul scent when bruised ( Figure 1). While the pungent smell has given rise to another of is common names ‘stinking roger’, it is considered to be a defence mechanism to prevent being eaten by herbivores such as cattle, an attribute that is reflected in the generic name *Ballota* which comes from the Latin ‘ballo’ meaning ‘to reject’.

The subspecies is found frequently in hedgerows and field-borders along walls on waste ground, and in disturbed sites, usually in the lowlands preferring nutrient-rich soils. It is frequent near habitations and is common in urban areas of England and Wales, but much rarer in Scotland and Ireland ( Botanical Society of Britain & Ireland, 2020). Outside Britain and Ireland it is also found across western, central and southern Europe, east to Central Asia, and has been introduced into Northeast Argentina, Cyprus, USA, and Sweden ( POWO, 2023).

While the species is probably not native to Britain and Ireland, it is considered to be an archaeophyte (i.e., introduced prior to c. 1500) as there is archaeological evidence suggesting that *B. nigra* has been associated with human settlements since the Iron Age ( Botanical Society of Britain & Ireland, 2020).

Like its relative, the white horehound ( *Marrubium vulgare*), *B. nigra* has a long history of use in herbal medicine, possibly going back as far as the 1st century CE, as it is reported that the *ballote* mentioned by Dioscorides in his ‘ *De Materia Medica’* (e.g., see translation by Osbaldeston and Wood, 2000) refers to *B. nigra* ( De Vos, 2010). Dioscorides suggested that leaves could be applied with salt as an antidote to being bitten by a rabid dog (possibly giving rise to another common name for *B. nigra* of ‘madwort’), while its application with honey could treat stinking ulcers. Interest in the species has continued to this day, with numerous reports investigating the secondary metabolites and their potential application for treating a range of medical conditions (e.g. see ( Przerwa *et al.*, 2020)

Here we present the first high-quality genome of *B. nigra* subsp. *foetida*, a diploid species with 2 *n* = 22 chromosomes. It joins the other 30 whole genome sequences in Lamiaceae published in peer-reviewed journals so far and is the first species to be sequenced from the tribe Marrubieae ( Usadel lab, 2023; Zhao *et al.*, 2021). Lamiaceae have the largest percentage of species with medicinal properties (13.7% of species; 1,059 out of 7,756 ( Willis, 2017)) out of all the flowering plant families, hence insights into its genome may be useful for enhancing research into the medicinal properties and their biosynthesis in this ancient herb.

## Genome sequence report

The genome was sequenced from a specimen of *Ballota nigra* ( Figure 1) from Royal Botanic Gardens, Kew, Surrey, UK (51.48, –0.3). Using flow cytometry, the genome size (1C-value) was estimated to be 1.45 pg, equivalent to 1,420 Mb. A total of 28-fold coverage in Pacific Biosciences single-molecule HiFi long reads and 55-fold coverage in 10X Genomics read clouds was generated. Primary assembly contigs were scaffolded with chromosome conformation Hi-C data. Manual assembly curation corrected 164 missing joins or misjoins and removed 2 haplotypic duplications, reducing the scaffold number by 85%, and increasing the scaffold N50 by 8.81%.

**Figure 2.**
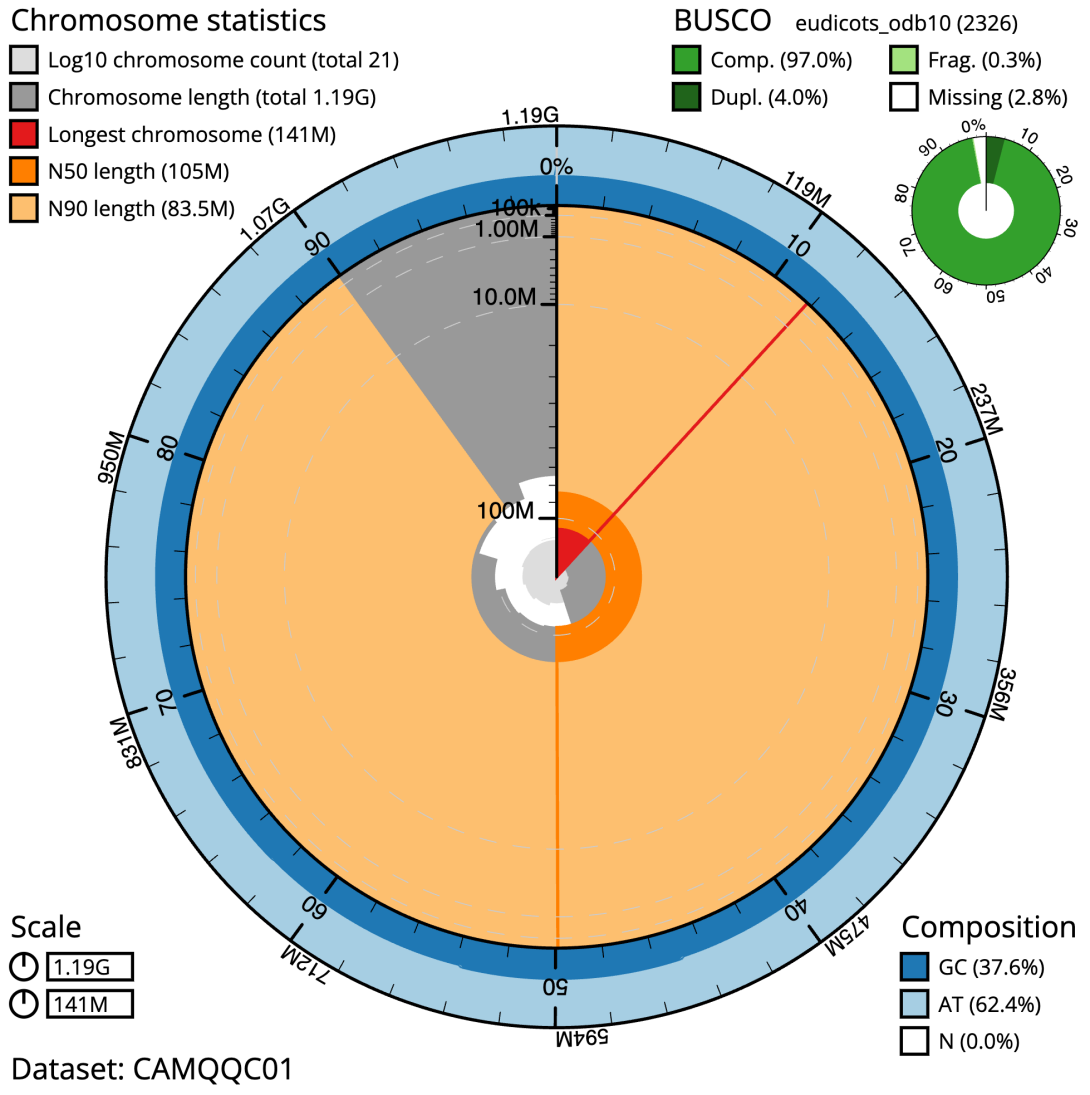
Genome assembly of *Ballota nigra*, daBalNigr1.1: metrics. The BlobToolKit Snailplot shows N50 metrics and BUSCO gene completeness. The main plot is divided into 1,000 size-ordered bins around the circumference with each bin representing 0.1% of the 1,187,337,993 bp assembly. The distribution of scaffold lengths is shown in dark grey with the plot radius scaled to the longest scaffold present in the assembly (140,828,634 bp, shown in red). Orange and pale-orange arcs show the N50 and N90 scaffold lengths (104,982,948 and 83,520,746 bp), respectively. The pale grey spiral shows the cumulative scaffold count on a log scale with white scale lines showing successive orders of magnitude. The blue and pale-blue area around the outside of the plot shows the distribution of GC, AT and N percentages in the same bins as the inner plot. A summary of complete, fragmented, duplicated and missing BUSCO genes in the eudicots_odb10 set is shown in the top right. An interactive version of this figure is available at https://blobtoolkit.genomehubs.org/view/daBalNigr1.1/dataset/CAMQQC01/snail.

The final assembly has a total length of 1,186.8 Mb in 17 sequence scaffolds with a scaffold N50 of 105.0 Mb ( Table 1). Most (99.94%) of the assembly sequence was assigned to 11 chromosomal-level scaffolds. Chromosome-scale scaffolds confirmed by the Hi-C data are named in order of size ( Figure 2– Figure 5; Table 2). A number of repetitive scaffolds were inserted to the left end of Chromosome 5. The order and orientation of these is unknown. While not fully phased, the assembly deposited is of one haplotype. Contigs corresponding to the second haplotype have also been deposited. The mitochondrial and plastid genomes were also assembled and can be found as contigs within the multifasta file of the genome submission.

**Table 1.** Genome data for *Ballota nigra*, daBalNigr1.1.

Project accession data		
Assembly identifier	daBalNigr1.1	
Species	*Ballota nigra*	
Specimen	daBalNigr1	
NCBI taxonomy ID	194200	
BioProject	PRJEB47666	
BioSample ID	SAMEA7522397	
Isolate information	daBalNigr1, leaves (DNA sequencing and Hi-C scaffolding)	
Assembly metrics *		*Benchmark*
Consensus quality (QV)	56.6	*≥ 50*
*k*-mer completeness	99.99%	*≥ 95%*
BUSCO **	C:97.0%[S:92.9%,D:4.0%], F:0.3%,M:2.8%,n:2,326	*C ≥ 95%*
Percentage of assembly mapped to chromosomes	99.94%	*≥ 95%*
Sex chromosomes	-	*localised homologous* *pairs*
Organelles	Three mitochondrial chromosomes and one chloroplast genome assembled.	*complete single alleles*
Raw data accessions		
PacificBiosciences SEQUEL II	ERR7012650–ERR7012652	
10X Genomics Illumina	ERR6895890–ERR6895893	
Hi-C Illumina	ERR6895894	
Genome assembly		
Assembly accession	GCA_947034835.1	
*Accession of alternate haplotype*	GCA_947034845.1	
Span (Mb)	1,186.8	
Number of contigs	245	
Contig N50 length (Mb)	11.7	
Number of scaffolds	17	
Scaffold N50 length (Mb)	105.0	
Longest scaffold (Mb)	140.8	

* Assembly metric benchmarks are adapted from column VGP-2020 of “Table 1: Proposed standards and metrics for defining genome assembly quality” from Rhie *et al.* (2021). ** BUSCO scores based on the eudicots_odb10 BUSCO set using v5.3.2. C = complete [S = single copy, D = duplicated], F = fragmented, M = missing, n = number of orthologues in comparison. A full set of BUSCO scores is available at https://blobtoolkit.genomehubs.org/view/daBalNigr1.1/dataset/CAMQQC01/busco.

**Figure 3.**
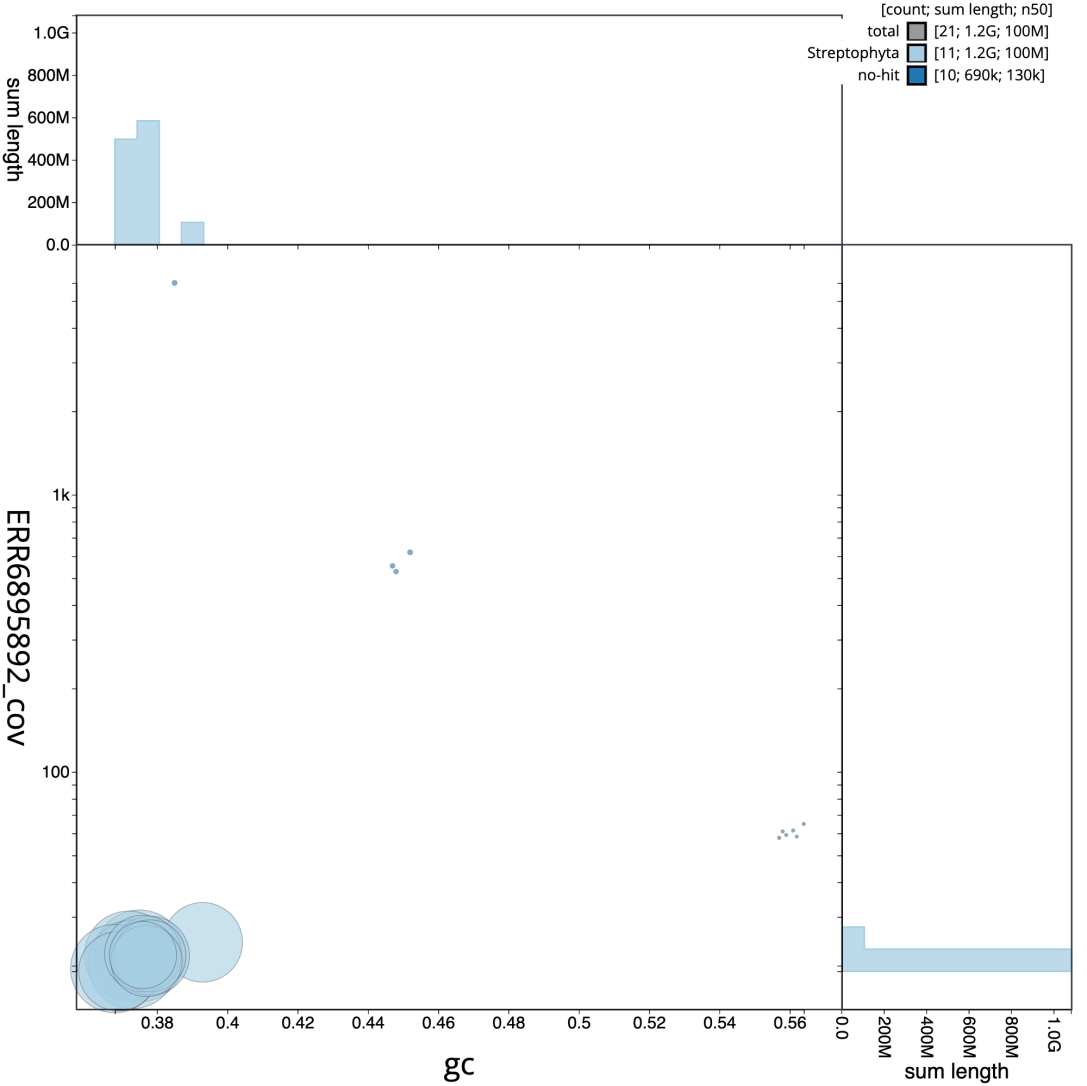
Genome assembly of *Ballota nigra*, daBalNigr1.1: BlobToolKit GC-coverage plot. Scaffolds are coloured by phylum. Circles are sized in proportion to scaffold length. Histograms show the distribution of scaffold length sum along each axis. An interactive version of this figure is available at https://blobtoolkit.genomehubs.org/view/daBalNigr1.1/dataset/CAMQQC01/blob.

**Figure 4.**
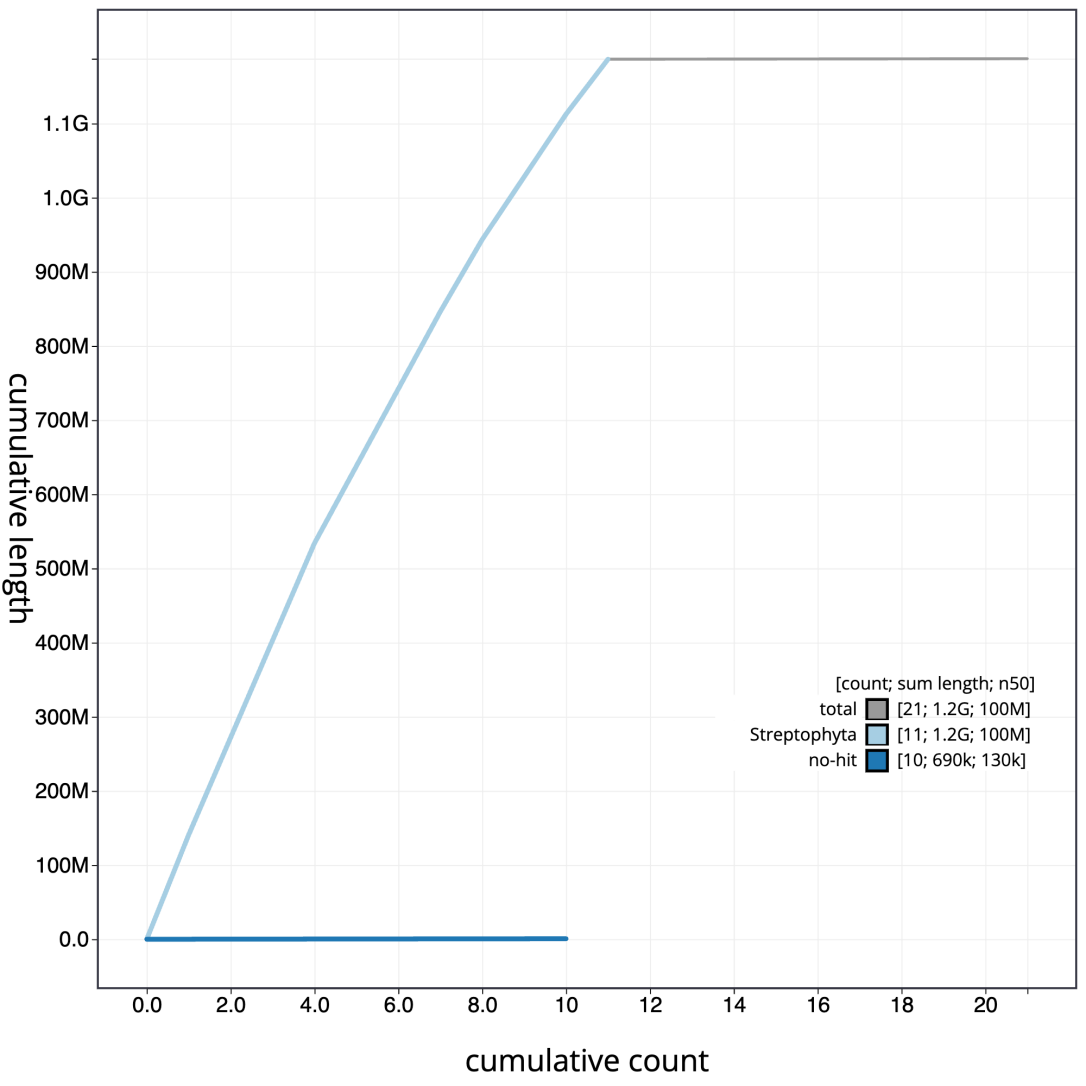
Genome assembly of *Ballota nigra*, daBalNigr1.1: BlobToolKit cumulative sequence plot. The grey line shows cumulative length for all scaffolds. Coloured lines show cumulative lengths of scaffolds assigned to each phylum using the buscogenes taxrule. An interactive version of this figure is available at https://blobtoolkit.genomehubs.org/view/daBalNigr1.1/dataset/CAMQQC01/cumulative.

**Figure 5.**
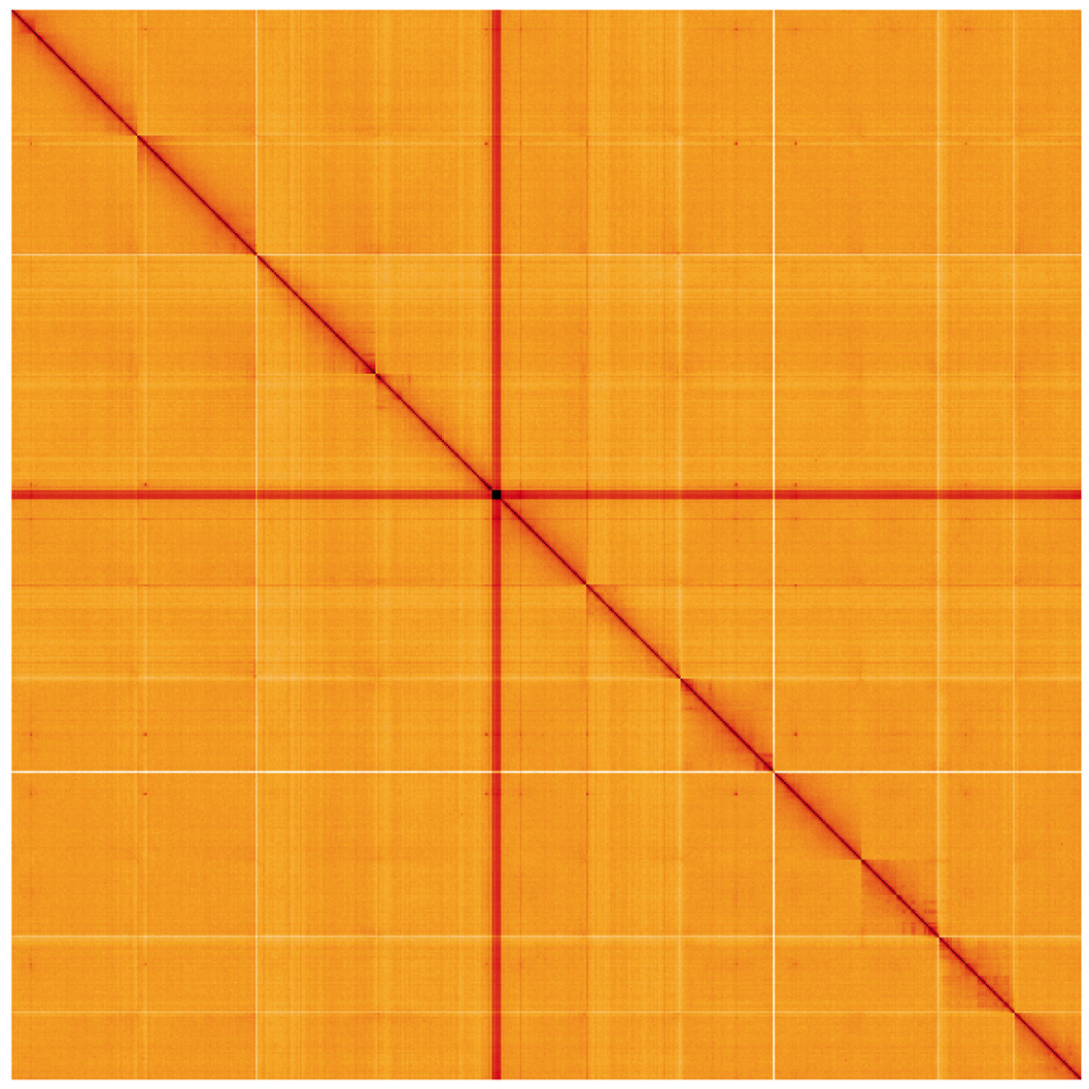
Genome assembly of *Ballota nigra*, daBalNigr1.1: Hi-C contact map of the daBalNigr1.1 assembly, visualised using HiGlass. Chromosomes are shown in order of size from left to right and top to bottom. An interactive version of this figure may be viewed at https://genome-note-higlass.tol.sanger.ac.uk/l/?d=Gm82rHCTTzWX0Ft7Bgitdw.

**Table 2.** Chromosomal pseudomolecules in the genome assembly of *Ballota nigra*, daBalNigr1.

INSDC accession	Chromosome	Length (Mb)	GC%
OX344720.1	1	140.83	37.5
OX344721.1	2	131.95	37.0
OX344722.1	3	131.2	37.5
OX344723.1	4	130.0	37.0
OX344724.1	5	104.98	39.5
OX344725.1	6	104.18	37.0
OX344726.1	7	103.09	38.0
OX344727.1	8	97.25	37.5
OX344728.1	9	85.53	38.0
OX344729.1	10	83.52	37.5
OX344730.1	11	74.12	37.5
OX344731.1	MT1	0.15	45.0
OX344733.1	MT3	0.13	45.0
OX344732.1	MT2	0.12	44.5
OX344734.1	Pltd	0.15	38.0

The estimated Quality Value (QV) of the final assembly is 56.6 with *k*-mer completeness of 99.99%, and the assembly has a BUSCO v5.3.2 completeness of 97.0% (single = 92.9%, duplicated = 4.0%), using the eudicots_odb10 reference set ( *n* = 2,326).

Metadata for specimens, spectral estimates, sequencing runs, contaminants and pre-curation assembly statistics can be found at https://links.tol.sanger.ac.uk/species/194200.

## Methods

### Sample acquisition, genome size estimation and nucleic acid extraction

A *Ballota nigra* (specimen ID KDTOL10077, ToLID daBalNigr1) was found growing in gravel beside a greenhouse in Royal Botanic Gardens, Kew, Surrey, UK (latitude 51.48, longitude –0.3) on 2020-09-02. The specimen was collected and identified by Maarten Christenhusz and frozen at –80°C.

The genome size was estimated by flow cytometry using the fluorochrome propidium iodide and following the ‘one-step’ method as outlined in Pellicer *et al.* (2021). Specifically for this species, the General Purpose Buffer (GPB) supplemented with 3% PVP and 0.08% (v/v) beta-mercaptoethanol was used for isolation of nuclei ( Loureiro *et al.*, 2007), and the internal calibration standard was *Petroselinum crispum* ‘Champion Moss Curled’ with an assumed 1C-value of 2,200 Mb ( Obermayer *et al.*, 2002).

DNA was extracted at the Tree of Life laboratory, Wellcome Sanger Institute (WSI). The daBalNigr1 sample was weighed and dissected on dry ice with tissue set aside for Hi-C sequencing. Leaf tissue was cryogenically disrupted to a fine powder using a Covaris cryoPREP Automated Dry Pulveriser, receiving multiple impacts. High molecular weight (HMW) DNA was extracted using the Qiagen Plant MagAttract DNA extraction kit. Low molecular weight DNA was removed from a 20 ng aliquot of extracted DNA using the 0.8X AMpure XP purification kit prior to 10X Chromium sequencing; a minimum of 50 ng DNA was submitted for 10X sequencing. HMW DNA was sheared into an average fragment size of 12–20 kb in a Megaruptor 3 system with speed setting 30. Sheared DNA was purified by solid-phase reversible immobilisation using AMPure PB beads with a 1.8X ratio of beads to sample to remove the shorter fragments and concentrate the DNA sample. The concentration of the sheared and purified DNA was assessed using a Nanodrop spectrophotometer and Qubit Fluorometer and Qubit dsDNA High Sensitivity Assay kit. Fragment size distribution was evaluated by running the sample on the FemtoPulse system.

### Sequencing

Pacific Biosciences HiFi circular consensus and 10X Genomics read cloud DNA sequencing libraries were constructed according to the manufacturers’ instructions. DNA sequencing was performed by the Scientific Operations core at the WSI on Pacific Biosciences SEQUEL II (HiFi) and Illumina NovaSeq 6000 (10X) instruments. Hi-C data were also generated from tissue of daBalNigr1 using the Arima2 kit and sequenced on the Illumina NovaSeq 6000 instrument.

### Genome assembly, curation and evaluation

Assembly was carried out with Hifiasm ( Cheng *et al.*, 2021) and haplotypic duplication was identified and removed with purge_dups ( Guan *et al.*, 2020). One round of polishing was performed by aligning 10X Genomics read data to the assembly with Long Ranger ALIGN, calling variants with FreeBayes ( Garrison & Marth, 2012). The assembly was then scaffolded with Hi-C data ( Rao *et al.*, 2014) using SALSA2 ( Ghurye *et al.*, 2019). The assembly was checked for contamination and corrected as described previously ( Howe *et al.*, 2021). Manual curation was performed using HiGlass ( Kerpedjiev *et al.*, 2018) and Pretext ( Harry, 2022). The mitochondrial genome was assembled using MitoHiFi ( Uliano-Silva *et al.*, 2022), which runs MitoFinder ( Allio *et al.*, 2020) or MITOS ( Bernt *et al.*, 2013) and uses these annotations to select the final mitochondrial contig and to ensure the general quality of the sequence.

A Hi-C map for the final assembly was produced using bwa-mem2 ( Vasimuddin *et al.*, 2019) in the Cooler file format ( Abdennur & Mirny, 2020). To assess the assembly metrics, the *k*-mer completeness and QV consensus quality values were calculated in Merqury ( Rhie *et al.*, 2020). This work was done using Nextflow ( Di Tommaso *et al.*, 2017) DSL2 pipelines “sanger-tol/readmapping” ( Surana *et al.*, 2023a) and “sanger-tol/genomenote” ( Surana *et al.*, 2023b). The genome was analysed within the BlobToolKit environment ( Challis *et al.*, 2020) and BUSCO scores ( Manni *et al.*, 2021; Simão *et al.*, 2015) were calculated.

Table 3 contains a list of relevant software tool versions and sources.

**Table 3.** Software tools: versions and sources.

Software tool	Version	Source
BlobToolKit	3.4.0	https://github.com/blobtoolkit/blobtoolkit
BUSCO	5.3.2	https://gitlab.com/ezlab/busco
FreeBayes	1.3.1-17-gaa2ace8	https://github.com/freebayes/freebayes
Hifiasm	0.15.3	https://github.com/chhylp123/hifiasm
HiGlass	1.11.6	https://github.com/higlass/higlass
Long Ranger ALIGN	2.2.2	https://support.10xgenomics.com/genome-exome/software/pipelines/latest/advanced/other-pipelines
Merqury	MerquryFK	https://github.com/thegenemyers/MERQURY.FK
MitoHiFi	2	https://github.com/marcelauliano/MitoHiFi
PretextView	0.2	https://github.com/wtsi-hpag/PretextView
purge_dups	1.2.3	https://github.com/dfguan/purge_dups
SALSA	2.2	https://github.com/salsa-rs/salsa
sanger-tol/genomenote	v1.0	https://github.com/sanger-tol/genomenote
sanger-tol/readmapping	1.1.0	https://github.com/sanger-tol/readmapping/tree/1.1.0

### Wellcome Sanger Institute – Legal and Governance

The materials that have contributed to this genome note have been supplied by a Darwin Tree of Life Partner. The submission of materials by a Darwin Tree of Life Partner is subject to the **‘Darwin Tree of Life Project Sampling Code of Practice’**, which can be found in full on the Darwin Tree of Life website here. By agreeing with and signing up to the Sampling Code of Practice, the Darwin Tree of Life Partner agrees they will meet the legal and ethical requirements and standards set out within this document in respect of all samples acquired for, and supplied to, the Darwin Tree of Life Project.

Further, the Wellcome Sanger Institute employs a process whereby due diligence is carried out proportionate to the nature of the materials themselves, and the circumstances under which they have been/are to be collected and provided for use. The purpose of this is to address and mitigate any potential legal and/or ethical implications of receipt and use of the materials as part of the research project, and to ensure that in doing so we align with best practice wherever possible. The overarching areas of consideration are:

•   Ethical review of provenance and sourcing of the material

•   Legality of collection, transfer and use (national and international)

Each transfer of samples is further undertaken according to a Research Collaboration Agreement or Material Transfer Agreement entered into by the Darwin Tree of Life Partner, Genome Research Limited (operating as the Wellcome Sanger Institute), and in some circumstances other Darwin Tree of Life collaborators.

## Data Availability

European Nucleotide Archive: *Ballota nigra*. Accession number PRJEB47666; https://identifiers.org/ena.embl/PRJEB47666. ( Wellcome Sanger Institute, 2022) The genome sequence is released openly for reuse. The *Ballota nigra* genome sequencing initiative is part of the Darwin Tree of Life (DToL) project. All raw sequence data and the assembly have been deposited in INSDC databases. The genome will be annotated using available RNA-Seq data and presented through the Ensembl pipeline at the European Bioinformatics Institute. Raw data and assembly accession identifiers are reported in Table 1. Members of the Royal Botanic Gardens Kew Genome Acquisition Lab are listed here: https://doi.org/10.5281/zenodo.4786680. Members of the Darwin Tree of Life Barcoding collective are listed here: https://doi.org/10.5281/zenodo.4893703. Members of the Plant Genome Sizing collective are listed here: https://doi.org/10.5281/zenodo.7994306. Members of the Wellcome Sanger Institute Tree of Life programme are listed here: https://doi.org/10.5281/zenodo.4783585. Members of Wellcome Sanger Institute Scientific Operations: DNA Pipelines collective are listed here: https://doi.org/10.5281/zenodo.4790455. Members of the Tree of Life Core Informatics collective are listed here: https://doi.org/10.5281/zenodo.5013541. Members of the Darwin Tree of Life Consortium are listed here: https://doi.org/10.5281/zenodo.4783558.
