# Supplementary material for: The genome sequence of black horehound, Ballota nigra L. subsp. foetida (Lam.) Hayek (Lamiaceae)
Source: Wellcome Open Res. 2023 Oct 12;8:439. [Version 1] doi: 10.12688/wellcomeopenres.19763.1 (PMC11499865; doi:10.12688/wellcomeopenres.19763.1)
Supplement: Peer review history

# Reviewer report: Reviewer response for version 1

**DOI:** 10.21956/wellcomeopenres.21889.r103832

**Published:** 2024-11-04

**Authors:** Parimalan Rangan

**Affiliations:** 1 ICAR-National Bureau of Plant Genetic Resources, New Delhi, India

1. Authors might use LAI metrics to assess the contiguity of the genome.  Alternatively, they can also use GCI tool to assess the assembly quality.

 2. The authors also might go for a structural annotation for the set of genes that the other researchers might be interested in, especially when it is a medicinal plant.

Are sufficient details of methods and materials provided to allow replication by others?

Yes

Is the rationale for creating the dataset(s) clearly described?

Yes

Are the datasets clearly presented in a useable and accessible format?

Yes

Are the protocols appropriate and is the work technically sound?

Yes

Reviewer Expertise:

Transcriptomics, Genomics, gene cloning, molecular biology

I confirm that I have read this submission and believe that I have an appropriate level of expertise to confirm that it is of an acceptable scientific standard.

# Reviewer report: Reviewer response for version 1

**DOI:** 10.21956/wellcomeopenres.21889.r103835

**Published:** 2024-10-23

**Authors:** Charlotte Lindqvist

**Affiliations:** 1 University at Buffalo, Buffalo, USA

In this note, the authors present a highly contiguous genome assembly of the lamioid species, *Ballota nigra*, that has a total length of 1,186.8 Mb in 17 scaffolds, with most of the assembly (99.94%) assigned to 11 chromosome-scale scaffolds. Despite the high number of species in the Lamiaceae family and their multiple medicinal uses and ecological importance, few genomes so far have been published, especially in the subfamily Lamioideae. This genome provides a useful resource for current and future research in the family.

**Minor comments:**

- The authors note that *B. nigra* subsp. *foetida* is a diploid species but there is no reference included or further mentioning of this. Was this confirmed with the assembled genome?
- In Table 1, it would be helpful to have a listing of the number of reads and/or base counts for each of the raw data types that went into the assembly (Pac Bio, 120X, and Illumina).
- The observed difference in estimated genome size based on flow cytometry (1,420 Mb) vs. assembled genome size (1,186.8 Mb) could be discussed, e.g., is this an indication of incompleteness of the genome assembly?
- Three mitochondrial genomes were assembled, and it would be helpful to know if any manual curation or quality checks were performed to confirm the reliability of these assemblies.
- The Hi-C contact map in Fig. 5 would be clearer if an outline of each chromosome was included and the highlighted red cross through the figure can probably be removed.
- Annotation of the genome and gene predictions would make this genome assembly even more useful.  

Are sufficient details of methods and materials provided to allow replication by others?

Yes

Is the rationale for creating the dataset(s) clearly described?

Yes

Are the datasets clearly presented in a useable and accessible format?

Partly

Are the protocols appropriate and is the work technically sound?

Yes

Reviewer Expertise:

Evolutionary genomics, plant phylogenetics

I confirm that I have read this submission and believe that I have an appropriate level of expertise to confirm that it is of an acceptable scientific standard.

# Reviewer report: Reviewer response for version 1

**DOI:** 10.21956/wellcomeopenres.21889.r96411

**Published:** 2024-09-20

**Authors:** Jian-Qiang Zhang

**Affiliations:** 1 Shaanxi Normal University, Xi'an, China

The authors have assembled a chromosome-level genome of *Ballota nigra* using PacBio HiFi and Illumina short reads, supplemented with Hi-C data. The genome is of high quality, with a long scaffold N50 and a high percentage of sequences assigned to chromosome-level scaffolds. The data presented in this study will be highly valuable for future research on Ballota nigra.

**Minor Comments:**

- The assembled genome size is smaller than the size obtained via flow cytometry. K-mer analysis could be used to survey the genome, including genome size, heterozygosity, etc.
- In Fig. 1, please add a scale bar to the figure.
- Please provide the amount of Hi-C data used to assist the assembly.
- In Fig. 2, the longest chromosome is should be 140.83 Mb.
- In Fig. 3, 'gc' could be revised to 'GC content of the genome (%)'.
- The LTR Assembly Index (LAI) could be used as an additional metric to assess the quality of the assembly.
- In Fig. 5, it would be better to outline each chromosome within a box for clarity

Are sufficient details of methods and materials provided to allow replication by others?

Yes

Is the rationale for creating the dataset(s) clearly described?

Yes

Are the datasets clearly presented in a useable and accessible format?

Yes

Are the protocols appropriate and is the work technically sound?

Yes

Reviewer Expertise:

Plant phylogenomics, speciation, biogeography

I confirm that I have read this submission and believe that I have an appropriate level of expertise to confirm that it is of an acceptable scientific standard.
